# A combined miRNA–piRNA signature to detect Alzheimer’s disease

**DOI:** 10.1038/s41398-019-0579-2

**Published:** 2019-10-07

**Authors:** Gaurav Jain, Anne Stuendl, Pooja Rao, Tea Berulava, Tonatiuh Pena Centeno, Lalit Kaurani, Susanne Burkhardt, Ivana Delalle, Johannes Kornhuber, Michael Hüll, Wolfgang Maier, Oliver Peters, Hermann Esselmann, Claudia Schulte, Christian Deuschle, Mathis Synofzik, Jens Wiltfang, Brit Mollenhauer, Walter Maetzler, Anja Schneider, Andre Fischer

**Affiliations:** 1 Department for Epigenetics and Systems Medicine in Neurodegenerative Diseases, German Center for Neurodegenerative Diseases (DZNE) Goettingen, 37075 Göttingen, Germany; 2 Translational Dementia Research, German Center for Neurodegenerative Diseases (DZNE), 53127 Bonn, Germany; 3 Bioinformatics Unit, German Center for Neurodegenerative Diseases (DZNE) Goettingen, 37075 Goettingen, Germany; 4 Department of Pathology and Laboratory Medicine, Boston University School of Medicine, Boston, Massachusetts, USA; 5 Department of Psychiatry and Psychotherapy, Friedrich-Alexander-Universität Erlangen-Nürnberg, 91054 Erlangen, Germany; 6 Center for Geriatric Medicine and Gerontology, University Medical Center Freiburg, 79106 Freiburg, Germany; 7 Department of Psychiatry and Psychotherapy, University Medical Centre Freiburg, 79106 Freiburg, Germany; 8 Department of Neurodegenerative Diseases and Geriatric Psychiatry, University of Bonn, 53127 Bonn, Germany; 9 Department of Psychiatry, Charité - Universitätsmedizin Berlin, corporate member of Freie Universität Berlin, Humboldt-Universität zu Berlin, and Berlin Institute of Health, 12200 Berlin, Germany; 10 German Center for Neurodegenerative Diseases (DZNE), 12203 Berlin, Germany; 11 Memory Clinic and Dementia Prevention Center, Experimental and Clinical Research Center (ECRC), 13125 Berlin, Germany; 12 Department of Psychiatry and Psychotherapy, University Medical Center Göttingen (UMG), 37075 Göttingen, Germany; 13 Center of Neurology, Department of Neurodegeneration and Hertie-Institute for Clinical Brain Research, University of Tuebingen, 72076 Tuebingen, Germany; 14 German Center for Neurodegenerative Diseases (DZNE), 72076 Tuebingen, Germany; 15 iBiMED, Medical Sciences Department, University of Aveiro, Aveiro, Portugal; 16 German Center for Neurodegenerative Diseases (DZNE) Goettingen, 37075 Göttingen, Germany; 17 Department of Neurology, University Medical Center Göttingen (UMG), 37075 Göttingen, Germany; 18 Paracelsus-Elena-Klinik, 34128 Kassel, Germany; 19 Department of Neurology, University Hospital Schleswig-Holstein, 24105 Kiel, Germany

**Keywords:** Epigenetics and behaviour, Psychiatric disorders

## Abstract

Alzheimer’s disease (AD) is the most common neurodegenerative disorder causing huge emotional and economic burden to our societies. An effective therapy has not been implicated yet, which is in part also due to the fact that pathological changes occur years before clinical symptoms manifest. Thus, there is a great need for the development of a translatable biomarker. Recent evidence highlights microRNAs as candidate biomarkers. In this study, we use next-generation sequencing to study the small noncoding RNAome (sncRNAome) in exosomes derived from human cerebrospinal fluid (CSF). We show that the sncRNAome from CSF-derived exosomes is dominated not only by microRNAs (miRNAs) but also by PIWI-interacting RNAs (piRNAs). We define a combined signature consisting of three miRNAs and three piRNAs that are suitable to detect AD with an AUC of 0.83 in a replication cohort and furthermore predict the conversion of mild–cognitive impaired (MCI) patients to AD dementia with an AUC of 0.86 for the piRNA signature. When combining the smallRNA signature with pTau and Aβ 42/40 ratio the AUC reaches 0.98. Our study reports a novel exosomal small noncoding RNA signature to detect AD pathology and provides the first evidence that in addition to miRNAs, piRNAs should also be considered as a candidate biomarker for AD.

## Introduction

Alzheimer’s disease (AD) is the most common reason of dementia in the elderly and is causing an increasing social–economic burden to our societies. AD arises on the pathological background of amyloid-beta deposition, the formation of neurofibrillary tangles, neuroinflammation, and neuronal cell death. Despite intensive research and an increasing understanding of the molecular processes that underlie AD^1–4^, an effective treatment has not been implemented so far, which is also due to the fact that suitable biomarkers that would allow the detection of pathology in the preclinical stage are still missing. This is of particular importance since pathological alterations occur years before the presentation of clinical symptoms^5^. Preclinical and prodromal disease stages are also the most promising time points for intervention trials. As such, there is great interest in molecular biomarkers that would help to identify patients early, predict the course of disease, and indicate therapeutic efficacy in clinical trials^6^. A recent line of research suggests that the analysis of circulating small noncoding RNAs (sncRNA) could serve as a diagnostic biomarker for various diseases^7^, including AD dementia^8^. A major focus has been on the analysis of microRNAs (miRNAs) that are 19–22-nucleotide-long RNA molecules regulating protein homeostasis via binding to a target mRNA thereby causing its degradation or inhibition of translation^9^. Recently, miRNAs have been implicated with learning and memory function^10–13^, and the pathogenesis of neurodegenerative diseases including AD^14–19^. For example, expression of specific miRNAs was altered in the brains of AD dementia patients and in AD animal models^11,14,16,17,20^, and preclinical data indicate that targeting miRNAs could help to reinstate protein homeostasis and memory function in AD^11^. Thus, miRNAs may offer an additional opportunity for patient stratification and therapy. Such approaches have been very promising in the field of oncology, and first-miRNA-based therapies are now in clinical testing. A number of studies have also investigated circulating microRNA expression in AD dementia, including the analysis of cerebrospinal fluids (CSF) by using PCR-based arrays, microarray, or more recently also next-generation sequencing approaches^20–24^. So far findings across studies are not consistent. Moreover, the source of small noncoding RNAs in CSF is not entirely clear. It has recently been shown that CSF miRNAs can be detected in exosomes^25–27^. Exosomes are cell-derived vesicles of 40–140-nm diameter that contribute to intercellular signaling and disposal of superfluous cellular content^28,29^. Exosomes have among other functions been implicated in neuronal plasticity.

Here, we use next-generation sequencing to quantify the smallRNA content in CSF exosomes in two independent cohorts of AD dementia patients and age-matched controls (Signature and replication cohort), and one prospective cohort of patients with mild–cognitive impairment (MCI) (DCN cohort). In contrast to miRNAs, other small noncoding RNA species have been rarely analyzed as candidate CSF biomarkers. The major sncRNA species detected were miRNAs and piwi-interacting RNAs (piRNA). We were able to identify a sncRNA signature consisting of three miRNAs and three piRNAs that distinguishes AD dementia patients from individuals without AD dementia with an AUC of 0.83. Importantly, sncRNAs were also able to predict the conversion of mild–cognitive impairment (MCI) patients to AD dementia with an AUC of 0.86. When combining the smallRNA data with CSF levels of Aβ 42, Tau, and pTau, we can detect AD patients and predict the conversion from MCI to AD dementia with AUC values of 0.98 and 0.97, respectively. We furthermore show that our signature can distinguish AD patients and non-demented control individuals when postmortem brain samples are analyzed. In conclusion, these data suggest that the analysis of exosomal smallRNAs, and especially piRNAs, could help to identify individuals at risk of developing AD dementia and improve the stratification of individuals to clinical trials.

## Methods

### Western blotting

Western blotting was performed according to standard protocols. Primary antibodies: mouse monoclonal antibodies against Flotillin-2 (BD Biosciences), Alix (BD Biosciences), TSG101 (GeneTex Inc., Irvine, CA, USA), CD-63 (BD Biosciences), and rabbit anti-Calnexin (StressGene). Secondary antibodies were obtained from Dianova and Invitrogen.

### Nanoparticle-tracking analysis

Exosomes in the ultracentrifugation pellet were analyzed by nanoparticle-tracking analysis with a NanoSight LM10 instrument and a LM14 viewing unit equipped with a 532-nm laser (NanoSight Ltd). Pellets from 100,000 × *g* centrifugation derived from 0.5 ml of total CSF were resuspended in 50 µl of PBS and diluted 1:40 in PBS. Samples were recorded in triplicates for 30 s. Particle numbers were then analyzed with the Nanoparticle Tracking Analysis (NTA) 2.3 software.

### Primary neuronal cell culture

Primary cortical and hippocampal neurons were prepared from E16 NMRI mouse embryos and cultured on poly-lysine-coated plastic dishes in serum-free MEM supplemented with B27 (Invitrogen) as described previously^30^. For exosome preparations, cells were cultured until day in vitro (DIV) 14. For exosome collection, cells were washed three times in phosphate-buffered saline (PBS) and incubated in fresh MEM B27 for 16 h. Culture medium was then collected and subjected to subsequent centrifugation steps performed at 4 °C: 3500 *×* *g* 10 min, 2 times 4500 *×* *g* for 10 min, 10,000 *×* *g* for 30 min, and 100,000 × *g* for 60 min. The 100,000 × *g* pellet was washed once with PBS before resuspension in sample buffer or Trizol. Parent cells were scraped into Trizol.

### Cerebrospinal fluid collection

**Fig. 1:**
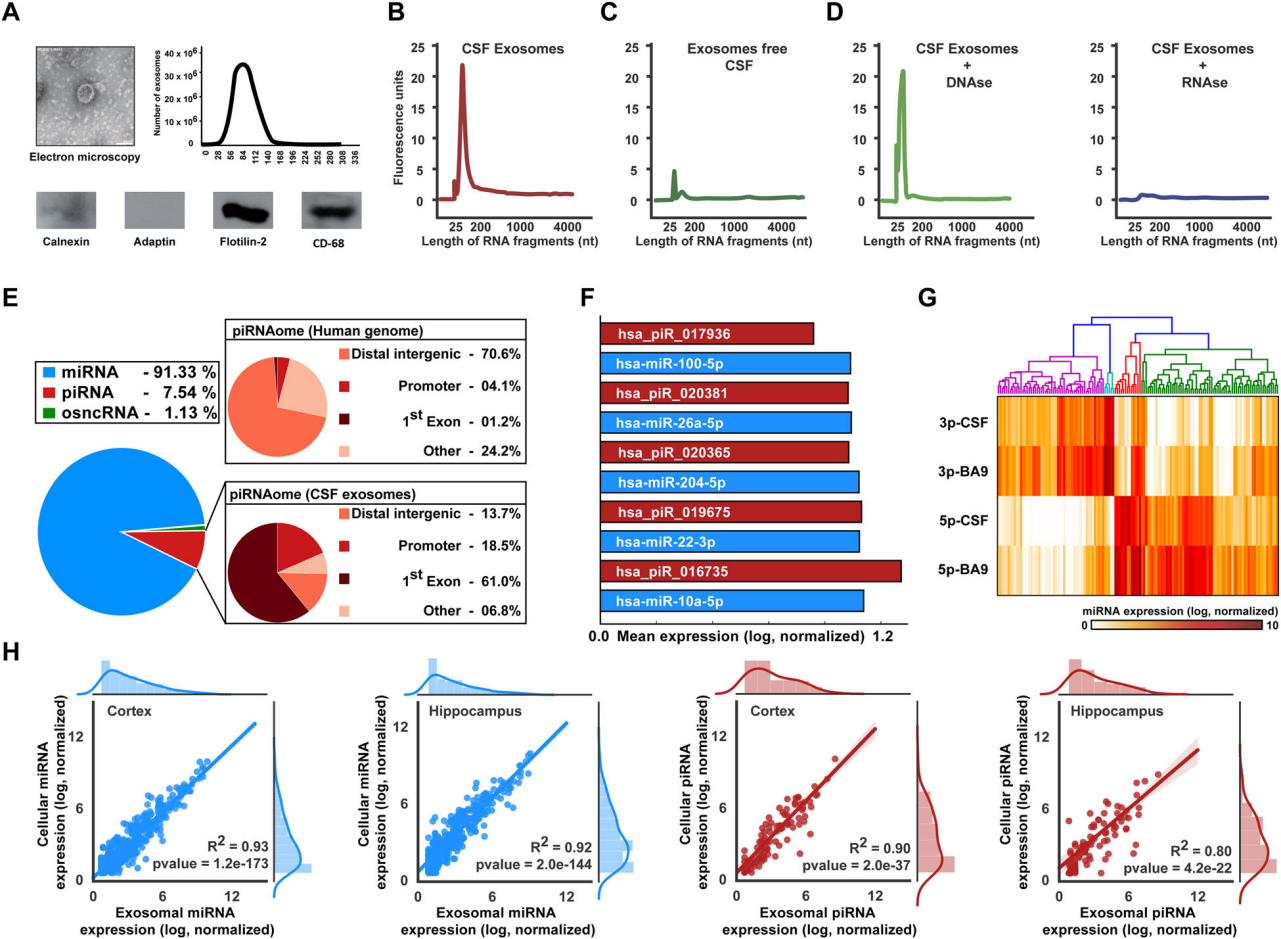
Analysis of the exosomal sncRNAome. **a** Exosomes isolated from human CSF were analyzed via EM (upper left panel), for fragment size by using a nanosight instrument (right panel) and via immunoblot for exosomal marker proteins (lower panel). **b** Electropherogram showing the profile of RNA isolated from exosomes. **c** Electropherogram showing the profile of RNA isolated from exosome-free CSF. **d** Electropherogram showing the profile of RNA isolated from lysed exosomes treated with DNAase (left) and RNAase (right). **e** Left panel: Pie chart showing the distribution of small noncoding RNAs in human CSF exosomes. Pie chart on the top right shows the genomic distribution of the human piRNAome for comparison. The lower right pie chart shows genomic annotation of the human CSF exosomal piRNAome. Note that in contrast to the entire piRNAome (upper right pie chart), the majority of piRNAs reside in the first exon of coding genes. **f** Top 5 expressed miRNAs (blue) and piRNAs (red) in human CSF exosomes. **g** Heatmap showing expression values of the 3-p and 5-p arms of all miRNAs detected in human CSF and in the human cortex (Brodmann Area 9). **h** Graphs showing Pearson correlation between miRNA (two left panels) and piRNA (two right panels) expression values of hippocampal and cortical neurons vs. the corresponding miRNA and piRNA expression in exosomes released from these cells

Human CSF samples (42 with Alzheimer’s dementia, 82 psychiatric and neurological controls, and 17 with MCI) were collected from the Department of Psychiatry at University Medical Center Göttingen (Germany), University Department of Neurology at University Hospital Tübingen (Germany), and Paracelsus Elena clinic Kassel (Germany) in two iterations between January 2012–March 2013 and April 2013–October 2014 with the approval of IRB at University Medical Center Göttingen (IRB 02/05/09), IRB approval by the local board of Hessen, Germany, IRB 09/07/04 and 26/07/02, at Paracelsus clinic Kassel and IRB approval 20/099/2011B01 for biobanking) at the Department of Neurology, Tübingen (Germany). After obtaining informed consent, ~10 ml of cerebrospinal fluid (CSF) were collected by lumbar puncture between 9 and 12 am. Thirty-eight randomly selected samples obtained from the neurological controls were used to confirm the presence of smallRNAs in CSF exosomes as described in Fig. 1. Specimens were collected in polypropylene tubes and centrifuged at 2000 *×* *g* for 10 min at room temperature (Göttingen and Kassel cohorts) or 4 °C (Tübingen cohort), aliquoted, and frozen at −80 °C within 30 min of completion of the procedure. All samples were obtained in accordance with the ethical standards laid down in the 1964 Declaration of Helsinki.

All AD dementia patients fulfilled the National Institute of Neurological and Communicative Disorders and Stroke and the Alzheimer’s Disease and Related Disorders Association (NINCDS-ADRDA) and National Institute on Aging and Alzheimer’s Association (NIAA) diagnosis criteria for probable AD dementia^31,32^. We employed CSF Aβ42 and CSF total Tau levels as fluid biomarkers in patients upon neuropsychological testing (AD was classified as Aβ42 < 450 [pg/ml] and total Tau > 200 [pg/ml]). Thus, AD pathology diagnosis was based on positive CSF Aβ42 and total Tau levels, whereas all controls were negative for these markers. Control CSFs included CSF from cognitively healthy patients with depression, cephalgy, schizophrenia, bipolar disorder, and polyneuropathy or were obtained from a cohort of healthy controls that had undergone neuropsychological testing to rule out cognitive impairment. None of the control patients suffered from neurodegenerative disorders or dementia. Based on these markers, we also defined two groups of MCI patients in the DCI cohort (see below): MCI due to AD and MCI control^33,34^. Only CSF samples with normal routine parameters were used. Samples with erythrocyte counts > 50/mm^3^ were excluded. CSF Aβ1-40/42, Tau, and phospho-tau (pTau) were determined with commercially available ELISA kits according to the manufacturer’s protocol (Innogenetics NV, Ghent, Belgium).

### The longitudinal DCN cohort (dementia competence network, DCN) of MCI patients

The Dementia Competence Network (DCN) cohort is a prospective multicenter observational study on memory clinic patients with MCI or early dementia. The DCN study was approved by the Ethics Review Board of the Erlangen medical faculty (coordinating center) and by the Ethics Committees at each individual center, and was conducted in accordance with the Declaration of Helsinki. The 10-year follow-up study was approved by the Ethics Committee of Göttingen University Medical center (IRB 40/7/02). All patients gave written informed consent to participate. Participants were recruited between 2003 and 2007 at 13 specialist memory clinics in Germany. For inclusion and exclusion criteria and a description of the cohort please refer to ref. ^35^. A MCI diagnosis was made on the basis of clinical and neuropsychological data, i.e., decline of cognitive abilities (>1 SD below age- and education-adjusted norms) in at least one of the domains of the Consortium to Establish a Registry of Dementia (CERAD) neuropsychological test battery and no changes in activities of daily living. We selected patients fulfilling MCI criteria at baseline and from which CSF was available and retrieved 10-year follow-up information either by telephone interview or a site visit of patient and caregiver to assess activities of daily living and cognitive functions. All participants on whom we could obtain 10-year outcome information, were included in our analysis (*n* = 17).

### Human brain tissue

Postmortem tissue from the prefrontal cortex (Brodmann area 9) from individuals that did not suffer from neurodegenerative diseases was obtained with ethical approval from the Alzheimer’s disease Research Center Brain bank at Massachusetts General Hospital, Boston, MA and from Brigham & Women’s Hospital Autopsy Service, Boston, MA, USA. Samples were matched for age and postmortem delay (*n* = 9, male 7, female = 2; age = 60 ± 19 years; PMD 16 ± 4 h).

### Neuropsychological procedures

All subjects were investigated with standardized neuropsychological tests as described previously^35^. The CERAD neuropsychological test battery includes verbal and visuspatial learning subtests with immediate and delayed recall, a naming test, a verbal fluency test, and the Mini Mental State Examination test (MMSE). We here focused on the verbal-delayed free-recall (CERAD-DR) measure. As a composite measure of overall dementia severity, the CDR sum-of-boxes (CDR-sb) was additionally applied in participants of the DNC cohort^36^. For comparison with other cohorts, the MMSE was applied^37^.

### Purification of exosomes from cerebrospinal fluid

Exosomes were isolated as described previously^38–40^ from 1 ml starting volume. CSF was thawn on ice and subjected to subsequent centrifugation steps at 4 °C: 3,500 × *g* for 10 min, two times 4,500 × *g* for 10 min, 10,000 × *g* for 30 min, and 100,000 × *g* for 60 min. The 100,000 × *g* pellet containing the exosomes was washed once with phosphate-buffered saline (PBS) at 100,000 × *g* for 60 min before resuspension in 200 µl of TRI Reagent® and stored at −80 °C for further use.

### Electron microscopy

Exosomes were prepared from CSF as described above. The 100,000 × *g* pellet was fixed with 4% paraformaldehyde and adsorbed to glow-discharged Formvar-carbon-coated copper grids by floating the grid for 10 min on 5-µl droplets on Parafilm. The grids were negatively stained with 2% uranyl acetate containing 0.7 M oxalate, pH 7.0, and imaged with a LEO EM912 Omega electron microscope (Zeiss, Oberkochen). Digital micrographs were obtained with an on-axis 2048 _ 2048 CCD camera (Proscan, Scheuring).

### RNA extraction and smallRNA sequencing

Fractions were stored at −80 °C until homogenization with 1 ml of TRI Reagent®, treated with 25 µl/ml of DEPC. After mixing with the 2 µl of glycogen, the mixture was kept at room temperature for 5 min. Two-hundred microliters of phenol–chloroform was added to the mix and kept at room temperature for further 5 min after vigorous shaking. After centrifugation for 15 min at 1200 × *g*, the aqueous phase containing RNA, was collected and incubated overnight in 500 µl of isopropanol at −20 °C. The precipitated RNA was subsequently isolated by 30 min of centrifugation at 12,000 × *g* at 4 °C. The RNA pellet was collected and washed two times with 75% ethanol. The RNA pellet was air dried and suspended in 10 µl of water. Smallrna libraries were prepared by using Illumina’s TruSeq® smallRNA kit following the manufacturer’s protocol.

### SmallRNAseq reads preprocessing

For processing of sequencing data, a customized in-house software pipeline was used. Illumina’s bcl2fastq (v 1.8.4) (Illumina, 2017) with default parameters was used to convert the base calls in the per-cycle BCL files to the per-read FASTQ format from raw images. Along with base calling, adapter trimming, removal of Unique Molecular Identifiers (UMIs), and demultiplexing were performed. Quality control of raw sequencing data was performed by using FastQC (v 0.11.5).

### Reads alignment and normalization

We first filtered out all the samples with library size (total uniquely mapped reads) <50,000 reads. We calculated miRNAs and piRNAs normalized counts by using Variance stabilization normalization (VSN)^41–43^. The resulting VSN counts were corrected for various cohorts along with the removal of the unwanted variances by using the *R* (v 3.2.2)^44^ package RUVSeq (v 1.14.0)^45^. We filtered out miRNAs and piRNAs that had a VSN read count less than 0.5 in the 95% of control and diseased samples, respectively. Thus, we obtained a set of 154 miRNAs and 43 piRNAs that were expressed and used for downstream analysis.

### Statistical and machine-learning analysis

In order to obtain a set of highly discriminative smallRNAs, i.e., smallRNAs that separate one cohort from another (e.g., controls from AD dementia), we used an iterative feature-selection approach based on the application of statistical and machine- learning techniques. In specific, the procedure consisted of three iterations that progressively trimmed down the initial set of sequenced smallRNAs up to the point of leaving a manageable signature for random forest classification. In iteration 1, we identified an initial subset of discriminatory smallRNAs by applying the Measure of Relevance (MoR) method^45^ in combination with reliability analysis (RiA), as described in ref. ^22^. The MoR method ranks the discrimination power of each smallRNA in an independent way, therefore yielding a reduced list of candidate smallRNAs. RiA is then subsequently applied as a control procedure to validate the results of MoR. As a result of applying Iteration 1, a reduced subset of smallRNAs was obtained. In iteration 2, the smallRNAs derived from iteration 1 were filtered out by using a machine-learning variable ranking method that is based on information-theoretic principles^46,47^. In iteration 3, we filtered out all miRNAs and piRNAs whose discriminative power was confounded by age and gender by applying the multivariate analysis of covariance (MANCOVA) analysis (Bonferroni corrected significance with *α* = 0.05) (Table S1).

The performance of the selected features was then evaluated on an independent test cohort. We used the random forest algorithm implemented in *R* (v 3.2.2) with the package randomforest (v 4.6.14). Input parameters like average error and the average number of trees were calculated by using a tenfold cross-validation on the training data. Then by using the optimized input parameters, a model is trained with stratified sampling and class weights (0.5, 1.0) for the control and the AD dementia class to minimize the false negatives. The prediction is performed on the replication cohort, and an AUROC (area under the receiver-operating characteristics) curve was used to estimate the performance from the untouched replication cohort data. The AUC values (with smoothing) were plotted by using the pROC package with 500 stratified bootstrap iterations along with the confidence interval for the AUC values.

## Results

### CSF exosomes contain sncRNAs linked to brain function

We started our analysis with the aim to confirm previous findings suggesting that small noncoding RNAs were present within CSF exosomes. To this end, we obtained CSF from 38 individuals that did not suffer from neurodegenerative diseases. Proper isolation of exosomes was confirmed via immunoblot analysis for the marker proteins Flotilin-2 and CD-63, electron microscopy, and via the analysis of particle size (Fig. 1a). Next, we analyzed RNA isolated from these exosomes and from the corresponding exosomal-free CSF fraction via a bioanalyzer microfluidic device. The corresponding electropherograms show that a significant amount of RNA with a particularly high peak indicating smallRNA species is detectable in the exosomal CSF fraction (Fig. 1b). In contrast, comparatively little RNA was obtained from the corresponding exosome-free CSF (Fig. 1c). Treating RNA samples obtained from CSF exosomes with DNAase did not affect RNA integrity (Fig. 1d), while treatment with RNAase eliminated the smallRNA peak (Fig. 1d). These data provide further evidence that human CSF contains exosomes that carry smallRNAs. Next we analyzed the RNA content of CSF exosomes via smallRNA sequencing. The smallRNA content was dominated by miRNAs and piRNAs (Fig. 1e). The five highest-expressed miRNAs in CSF exosomes were miR-10a-5p, miR-100-5p, miR-22-3p, mIR-204-5p, and miR-26a (Fig. 1f) that have been previously linked to memory function and/or neurological diseases^27,48,49^ (Fig. 1f). The five top-expressed piRNAs showed comparable expression levels to miRNAs (Fig. 1f). In contrast to miRNA, the role of piRNA is not well established, but there is evidence that in addition to a role in silencing repetitive genomic regions thereby mediating genomic stability, some piRNAs are believed to play an active role in gene-expression control^50^. It is thus interesting that the piRNAs detected in the CSF exosomes are mainly expressed from the first exon of a host gene (Fig. 1e), a pattern that differs dramatically when compared with all piRNAs encoded in the human genome that are mainly expressed from intergenic regions (Fig. 1e). Regarding the microRNAome, the 5- and the 3-p arm of a given miRNA are often expressed at different levels. Since for many microRNAs, the precise biological function is still unclear, a suitable estimate to define if the 5-p or the 3-p arm of a given miRNA is biologically active, is to quantify the expression of both arms and consider the highest expressed arm to be the active one. It was previously suggested that the inactive arm of a given microRNA would be sorted into exosomes as a cellular-clearance mechanism. At the same time, there is evidence that exosomal transport of molecules from one cell to another via exosomes serves important biological functions^51^. To evaluate these possibilities in the context of exosomal miRNAs from CSF, we performed smallRNA sequencing from postmortem human prefrontal cortex (Brodmann area 9, individuals without neurological disease) and compared the expression pattern of the 5-p and 3-p arms for all miRNAs detected in CSF exosomes with the corresponding expression pattern observed in human brain. The pattern of the 3-p vs. 5-p arms of microRNAs detected in CSF was similar to the pattern observed in postmortem human brain tissue and confirmed that for the majority of the miRNAs only one arm was highly expressed (Fig. 1g). This finding supports the view that the miRNA content of CSF exosomes—at least in part—resembles the miRNA content of the parental cell. These data have to be interpreted with care, however, since the parental cell for CSF exosomes is not precisely known and likely includes various brain regions and also non-neural cells^25^. To further address the question if the miRNA and piRNA content of exosomes released from neurons would allow to make conclusions about the corresponding cellular sncRNAome, we decided to test the correlation of miRNA and piRNA expression in primary cortical and hippocampal neurons and in their corresponding exosomes. Exosomes were isolated from the media supernatant of donor cells. Subsequently, the exosomal and the cellular RNA were prepared and subjected to smallRNA sequencing. For both, miRNAs and piRNAs, we detected a highly significant correlation between cellular and exosomal fractions (Fig. 1h).

These data suggest that exosomes released from neurons mainly represent the sncRNA composition of the parental cells, a finding that is in line with other studies performed, for example, on tumor cells^52–54^. It is therefore possible that the sncRNAome of CSF exosomes—at least in part—reflects the scnRNAome of neuronal and non-neuronal cells of the adult brain.

### A CSF small noncoding RNA signature to diagnose AD dementia patients

**Fig. 2:**
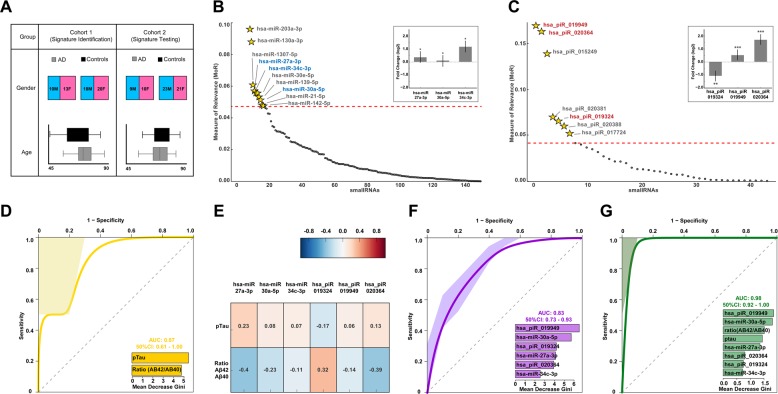
A sncRNA signature to diagnose AD patients. **a** Demographic information of the human cohorts used for signature identification and testing. **b** Measure of Relevance (MoR) analysis for miRNA differences between AD and control samples in the signature cohort. The dotted red line represents the critical MoR value cut off. miRNAs above the dotted red line are considered informative. miRNAs marked in blue are not confounded by age and gender after MANCOVA analysis. The inset shows fold-change (log2) value of the three identified miRNAs between control and AD patients. Their significance level (Bonferroni-corrected *p* value < = 0.05) with their significance codes: 0 ‘***’ 0.001 ‘**’ 0.01 ‘*’ 0.05 ‘.’ 0.1 ‘’ 1 is shown on the top of the bars. **c** Measure of Relevance (MoR) analysis for piRNA differences between AD and control samples in the signature cohort. The dotted red line represents the critical MoR value cutoff. piRNAs above the dotted red line are considered informative. piRNAs marked in red are not confounded by age and gender after MANCOVA analysis. The inset shows the fold-change (log2) value of the three identified piRNAs between control and AD patients. Their significance level (Bonferroni-corrected *p* value ≤0.05) with their significance codes: 0 ‘***’ 0.001 ‘**’ 0.01 ‘*’ 0.05 ‘.’ 0.1 ‘ ’ 1 is shown on the top of the bars. **d** Receiver-operating characteristic (ROC) plot was obtained during the performance testing by using pTau levels and Aβ42/40 ratio obtained from CSF samples of the signature cohort on the replication cohort. Training was performed on the signature cohort with a tenfold cross-validation. The inset plot shows the variable importance. **e** Heatmap showing Pearson correlation coefficient between normalized expression of the sncRNA signature, pTau, and Aβ42/40 ratio in the signature cohort. Note that the sncRNA signature does not correlate significantly with *p*Tau levels and Aβ42/40 ratio. **f** ROC showing performance of the six sncRNA signatures when tested on the replication cohort. Training was done on the signature cohort with a tenfold cross-validation. The inset plot shows the variable importance of the six individual sncRNAs. **g** ROC showing performance of the combined 6 sncRNA signatures with pTau and Aβ42/40 ratio levels on the replication. A mean AUC of 0.98 was obtained. F, female; M, male

**Fig. 3:**
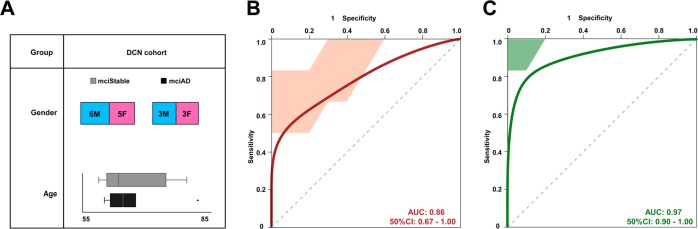
A sncRNA signature to predict conversion of MCI patients. **a** Demographic information of the DCN longitudinal and multicenter cohort of MCI. In total, baseline CSF exosomes from 17 participants diagnosed with MCI were analyzed. At the 10-year follow-up six individuals had converted to dementia (3 male and 3 female). **b** ROC shows performance of our three piRNA signatures (identified in the signature cohort) on the DCN cohort consisting of converting and stable MCI participants. **c** ROC with the mean AUC of 0.96 was obtained by using a combination of our piRNA signature with pTau and Aβ42/40 ratio values. Error bars indicate SD

Next, we investigated the miRNA and piRNA expression from CSF exosomes in two independent cohorts of AD dementia patients and controls. Cohort 1 (signature identification cohort) consisted of 23 AD dementia patients and 38 control individuals that did not suffer from any neurodegenerative disorder (Fig. 2a). Samples were collected at the University Medical Center Göttingen (Germany), Department of Psychiatry, Göttingen (Germany), and Department of Neurology at University Tübingen (Germany) between January 2012 and March 2013. The replication cohort (Cohort 2, signature-testing cohort) consisted of 19 AD dementia cases and 44 control individuals from which CSF samples were collected at University Medical Center Göttingen (Germany), Department of Psychiatry, Göttingen (Germany), Department of Neurology at University Tübingen (Germany), and Paracelsus Elena clinic Kassel (Germany) between April 2013 and October 2014 (Fig. 2a). We decided to employ cohort 1 for the identification of a smallRNA signature suitable to diagnose AD, while cohort 2 was used to test the performance of such a signature in an independent replication cohort (Fig. 2a). We employed an iterative approach to obtain an informative set of expressed and relevant features (miRNAs and piRNAs) by using MoR^55^, reliability analysis (RiA)^22^, machine-learning variable ranking^46,47^, and multivariate analysis of covariances on cohort 1 to generate a model that would be able to classify AD dementia patients and control individuals. We detected 3 miRNAs (Fig. 2b) and 3 piRNAs (Fig. 2c) as the most relevant features that were not confounded by age and gender (see also supplementary table 1). The identified miRNAs were miR-27a-3p, miR-30a-5p, and miR-34c (Fig. 2b) that have all been linked to memory function and neurodegeneration in previous studies^11,21,56,57^. The three miRNAs were increased in AD dementia patients (Fig. 2b). Regarding the piRNAs, piR_019324 was decreased, while piR_019949 and piR_020364 were increased in the analyzed CSF samples of AD dementia patients (Fig. 2c). While AD dementia patients used in this study were additionally diagnosed on the basis of CSF Aβ42 and CSF total Tau levels as fluid biomarkers in patients upon neuropsychological testing, for all participants of Cohort 1 and 2 measures for the CSF biomarker pTau and Aβ40 were also available. Thus, we used pTau and the Aβ42/40 ratio to train a model on cohort 1 that was then tested on cohort 2 by using a tenfold CV random forest algorithm. As expected, the combination of pTau levels and the Aβ42/40 ratio were able to classify AD dementia patients with high sensitivity and specificity (Fig. 2d). Of note, almost none of the identified miRNAs and piRNAs were significantly correlated to pTau or Aβ42/40 ratio levels, indicating that these smallRNAs do not simply reflect changes in pTau or amyloid pathology (Fig. 2e). The performance of our sncRNA model was then tested on the replication cohort by using a tenfold CV random forest algorithm. The miRNA/piRNA signature was able to distinguish AD dementia patients from controls with an AUC of 0.83 that was similar to that of pTau and Aβ42/40 ratio (Fig. 2f). Since our data indicated that the information encoded in the miRNA/piRNA signature does not simply reflect changes in Tau and amyloid pathology, we decided to combine the measures of pTau and Aβ42/40 ratio and our miRNA/piRNA signature. By this approach, we were able to classify AD dementia patients in the replication cohort correctly in 98% of the cases (Fig. 2g).

### A piRNA signature to predict conversion from MCI to AD dementia

Our data suggest that the analysis of miRNAs and piRNAs in CSF exosomes can improve the antemortem diagnosis of AD and might therefore also help to stratify patients for clinical trials. From a therapeutic point of view, it is of importance to develop a biomarker that could also help to predict the conversion of patients suffering from mild–cognitive impairment (MCI) to AD dementia. To address this question, we obtained CSF exosomes from individuals that were diagnosed with MCI and subjected to CSF collection between 2003 and 2004 (DCN cohort). Ten years later, 6 individuals had progressed to AD dementia, while 11 patients had developed stable MCI (Fig. 3a). Next, we tested if our miRNA/piRNA signature (see Fig. 2) would be able to also distinguish stable MCI patients from those that would convert to AD dementia in the DCN cohort. While the combined miRNA/piRNA signature had only limited predictive value, we noticed that this was due to the miRNAs (Fig. S1). Interestingly, we observed that the piRNA signature could predict conversion from MCI to AD with an AUC of 0.86 (Fig. 3b). Notably, when we combined the analysis of the piRNA signature with measures of pTau and the Aβ42/40 ratio, we were able to predict conversion from MCI to AD dementia with an AUC of 0.96 (Fig. 3c). In contrast, combined measures of pTau and the Aβ42/40 ratio predicted the progression to AD dementia with a comparatively lower AUC of 0.59 (Fig. S1C).

### A miRNA/piRNA signature is able to classify AD dementia patients on the basis of postmortem brain tissue

**Fig. 4:**
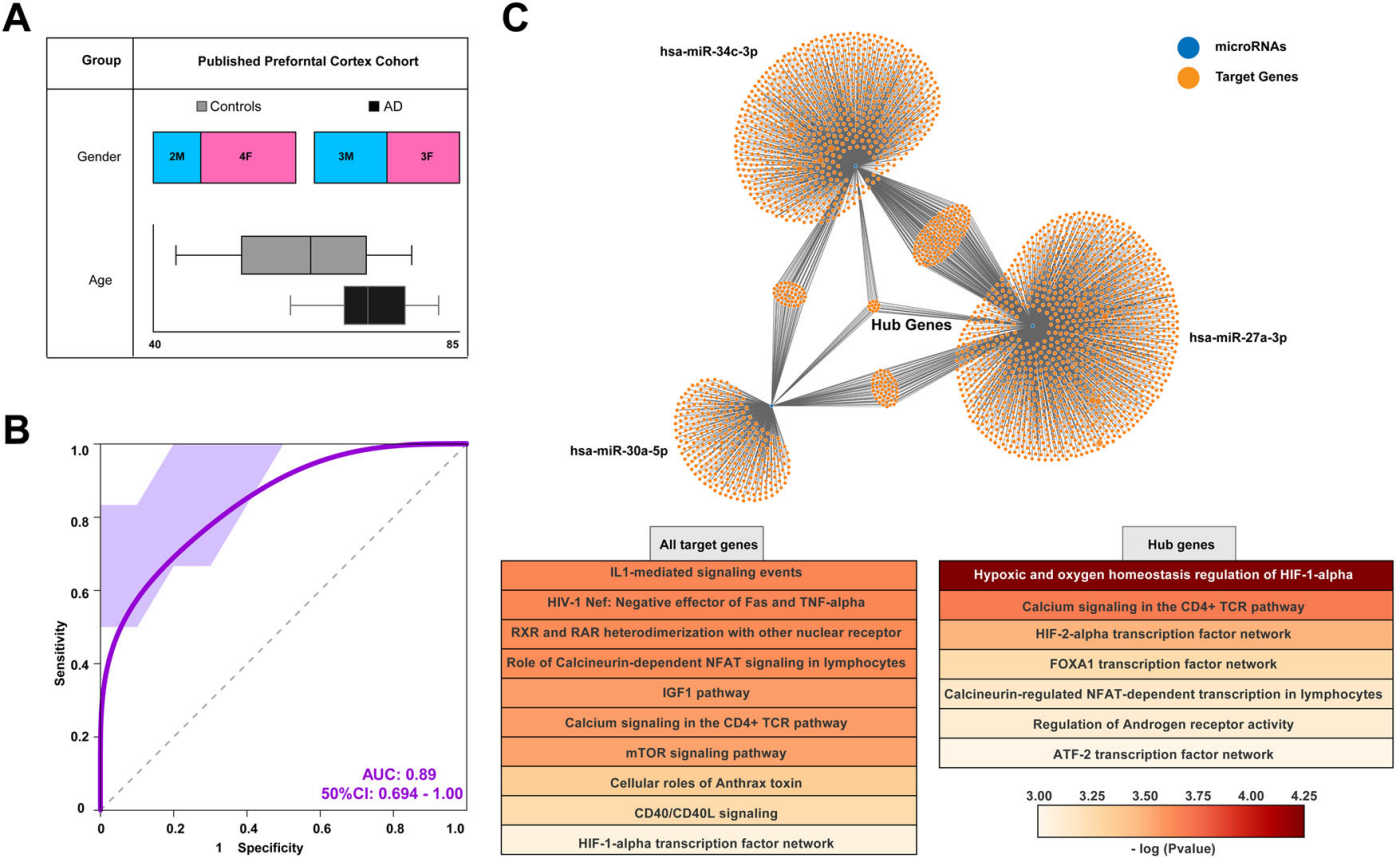
Performance of the CSF sncRNA signature in classifying patients on the basis of postmortem brain tissue. **a** Demographic information for postmortem brain tissue samples included in the analysis (published dataset GSE48552). **b** The six sncRNA signatures defined via the analysis of the signature cohort were tested on the data obtained from postmortem brain tissue (published brain cohort). ROC reveals a mean AUC of 0.89 suggesting that the sncRNA signature obtained from CSF helps to diagnose AD patients based on sncRNA expression in postmortem brain tissue. **c** Upper panel shows the confirmed target genes of the three miRNAs that are part of our sncRNA signature. Lower panel shows the significantly enriched signaling pathways based on the confirmed targets of the three miRNAs that are a part of the sncRNA signature. Error bars indicate SD

The finding that our miRNA/piRNA signature helps to diagnose AD and may also help predict conversion of MCI to AD dementia in independent replication cohorts is particularly interesting. To gain first insight, if the identified miRNA/piRNA signature may also inform about the relevant pathomechanisms in the brain, we decided to employ a published dataset in which smallRNAome analysis had been performed on postmortem human brain tissue (prefrontal cortex) from AD dementia patients and age-matched controls (Fig. 4a; *P* = 0.7 for age between AD and control, ANOVA)^58^. We then asked if our miRNA/piRNA signature would be able to classify AD dementia patients and controls on the basis of the sequencing dataset available from these postmortem human brains. Our analysis revealed that our signature was able to classify AD dementia patients and controls with an AUC of 0.89 (Fig. 4b). This finding indicates that the CSF exosomal miRNA/piRNA signature might inform—at least in part—about the pathomechanisms in the brain. We therefore also performed a pathway analysis of the confirmed target genes on the three microRNAs. When we analyzed the pathways linked to the confirmed target genes of all three microRNAs, we identified pathways highly relevant to AD, namely pathways linked to inflammatory processes, to IGF1 and mTOR signaling, and to HIF1alpha-related hypoxia^59–62^ (Fig. 4c). A number of genes were targeted by all three identified microRNAs and we named these genes “hub genes” (Fig. 4c). When we analyzed the predicted targets of these “hub genes” the most pronounced pathway was related to HIF1alpha-mediated hypoxia, followed by pathways linked to inflammatory processes and regulation of androgen receptor activity that has also been recently linked to AD^63^ (Fig. 4c).

In contrast to miRNAs, the role of piRNA is less well established. While the majority of piRNAs in the human genome are expressed from intergenic regions (see Fig. 1), the CSF-expressed piRNAs, including the three piRNAs of our signature, are mainly expressed from the first exon of coding genes. The corresponding genes are listed in Supplementary Table 2 (Table S2) and include genes linked to Alzheimer’s disease such as phospholipase A2^64^. In conclusion, these data further support the view that the identified miRNA/piRNA signature might reflect changes in pathways highly relevant to AD pathogenesis.

## Discussion

Several studies suggest that the analysis of the miRNAome could be a suitable approach for biomarker detection in various diseases, including disorders of the central nervous system^8,65,66^. Here we investigated the hypothesis that miRNAs found in CSF exosomes may inform about the pathogenesis of AD. We specifically focused our analysis on CSF-derived exosomes, since at least some of these exosomes likely stem from brain cells and may thus reflect pathological changes occurring in the brain. We detected miRNAs via smallRNA sequencing of CSF-derived exosomes, which is in agreement with two previous studies reporting the analysis of miRNAs obtained from human CSF exosomes via PCR arrays^27,24^. Another recent study reported successful smallRNA sequencing from human CSF exosomes isolated from three healthy donors^67^. In line with our observation, this study also suggests a specific enrichment of miRNAs in CSF exosomes when compared with the exosome-free supernatant. The same study also reported a significant overlap of miRNAs detected in CSF exosomes and the human brain. This is in line with our comparative analysis of human CSF exosomes and human postmortem brain tissue, indicating that the CSF exosomal miRNAome—at least in part—reflects the CNS microRNAome. While the precise role of CSF-derived exosomal miRNA remains to be elucidated, there is recent evidence suggesting, for example, a functional role of exosomal-released microRNAs in brain aging^68^. Nevertheless, CSF-derived exosomes likely also stem from other sources than the brain, and specific methods to enrich brain-derived exosomes from CSF before sequencing will be necessary to provide conclusive answers. We detected 3 miRNAs as part of a six-sncRNA signature that helped to diagnose and predict AD dementia, namely miR-34c, miR-30a, and miR-27a. It is interesting that all three miRNAs have been linked to AD via independent studies underscoring the importance of our observation. To this end, miR-34c was found to be upregulated in the hippocampus of mouse models for amyloid deposition and age-associated memory decline^11^. These data were confirmed in human postmortem brain tissue from AD dementia patients^11,21^. In addition, inhibition of miR-34c function ameliorated memory impairment and gene expression in a mouse model for amyloid deposition^11^. Interestingly, miR-34c was found to be elevated in response to pathological stress^69^, which is in line with data showing that stress-related diseases increase the risk for developing dementia via aberrant gene activity^70^. These data are also interesting since miR-30a expression was associated with altered neuropeptide Y signaling—that is intimately linked to stress-related neuropsychiatric diseases^56^. Moreover, miR-30a was identified as a differentially expressed miRNA in CSF from AD dementia patients in an early study comparing microRNAs in 9 controls and 7 AD dementia patients (Braak and Braak stage 5) via PCR array^20^. Another previous study reported altered levels of miR-27a in CSF from AD dementia patients and suggested the analysis of this miRNA as a candidate biomarker^57^. It has to be noted that while the increased expression of miR-34c and miR-30a is in line with the previously reported expression data, miR-27a was found to be decreased in CSF from AD dementia patients^57^. This discrepancy to our findings may stem from the fact that CSF exosomes were analyzed in our study, and that, so far all available data represent cross-sectional analysis. Thus, we speculate that dynamic changes in miRNA expression may be observed when patients are analyzed longitudinally. Another important consideration is the fact that sample size of the employed cohorts should be increased. It has to be mentioned that some previous studies have analyzed microRNAs in CSF of AD dementia patients by using different detection methods such as PCR array^20–24^. The data among these studies and our work are variable. One possible explanation results from a recent work suggesting that contamination of CSF with blood cells is a major confounding factor when analyzing CSF miRNAs^71^. In such a scenario, the analysis of cell-free CSF exosomes could be superior to the analysis of total CSF and may also explain some of the discrepancies among data. Moreover, different disease stages of the analyzed patients could also explain differences in miRNA levels, and most studies—including ours—are characterized by comparable small sample size, and especially for the DCN cohort, we cannot exclude a selection bias during the 10-year follow-up analysis.

In addition to miRNAs we detected a substantial amount of piRNAs in CSF exosomes. To the best of our knowledge, this is the first report that piRNAs are differentially expressed in human CSF exosomes of AD dementia patients. Our data are, however, in line with a recent report suggesting that piRNAs might play an important role in AD pathology, and more specifically that Tau pathology disrupts piRNA function leading to genome instability^72^. Moreover, loss of genome stability is emerging as an important process in AD pathology^73,72^ and it is thus possible that the observed changes in CSF piRNAs reflect the degree of neural genome instability. In addition, piRNAs have been also associated with the active regulation of gene expression in response to relevant stimuli, and changes in piRNAs may thus also reflect changes in cellular signaling. When compared with microRNAs, the role of piRNAs is however less well understood, and since piRNAs are also only poorly conserved among species^74^, bioinformatic prediction of their function is difficult at present. Future research needs to address the role of the 3 piRNAs identified in our study in human neuronal cells. Nevertheless, we speculate that the analysis of miRNAs and piRNAs is especially suitable as a candidate biomarker for complex diseases such as AD, since changes in these sncRNA signatures may reflect subtle changes in various signaling pathways critical for cellular homeostasis^65^. In line with this idea, the pathway analysis of the confirmed target genes from the three miRNAs of our signature indicates neuroinflammatory responses, altered IGF signaling, hypoxia, and mTOR signaling, all processes that are intimately linked to AD pathology^59,61,62^. As such, the analysis of sncRNA in CSF exosomes likely informs about multiple pathological processes that—when analyzed individually—would not be able to classify patients.

An important issue is also the identification of predictive biomarkers that inform about the future conversion to AD dementia. Recent advances suggest, for example, that the analysis of neurofilament light-chain protein in CSF is an early indicator of neuronal cell death in individuals suffering from familial AD dementia^75^. It is thus interesting that our reported piRNA signature was able to predict conversion of MCI patients to dementia. Nevertheless, these data have to be interpreted with care, and larger cohorts need to be analyzed in future studies.

In conclusion, our data report a pi/miRNA signature that helps to detect AD on the basis of CSF samples, and may also help to predict conversion of MCI patients. Our sncRNA signature reflects multiple homeostatic changes in various cellular processes and thus further improves the performance of established CSF biomarkers such as the analysis of amyloid peptides and Tau protein. Since smallRNAs are also very stable in cell-free environments, are resistant to thaw–freeze cycles, and are less prone to confounding factors related to the quantification method, we suggest that the analysis of miRNAs/piRNAs and the identified signature reported here in particular should be further considered as a candidate biomarker for AD stages.

## Supplementary information

Supplemental figure 1

Supplemental table 1

Supplemental table 2
