# Supplementary material for: A combined miRNA–piRNA signature to detect Alzheimer’s disease
Source: Transl Psychiatry. 2019 Oct 7;9:250. doi: 10.1038/s41398-019-0579-2 (PMC6779890; doi:10.1038/s41398-019-0579-2)
Supplement: Supplementary file 1 — Supplemental figure 1 [file 41398_2019_579_MOESM1_ESM.docx]

**Supplemental Figure 1**


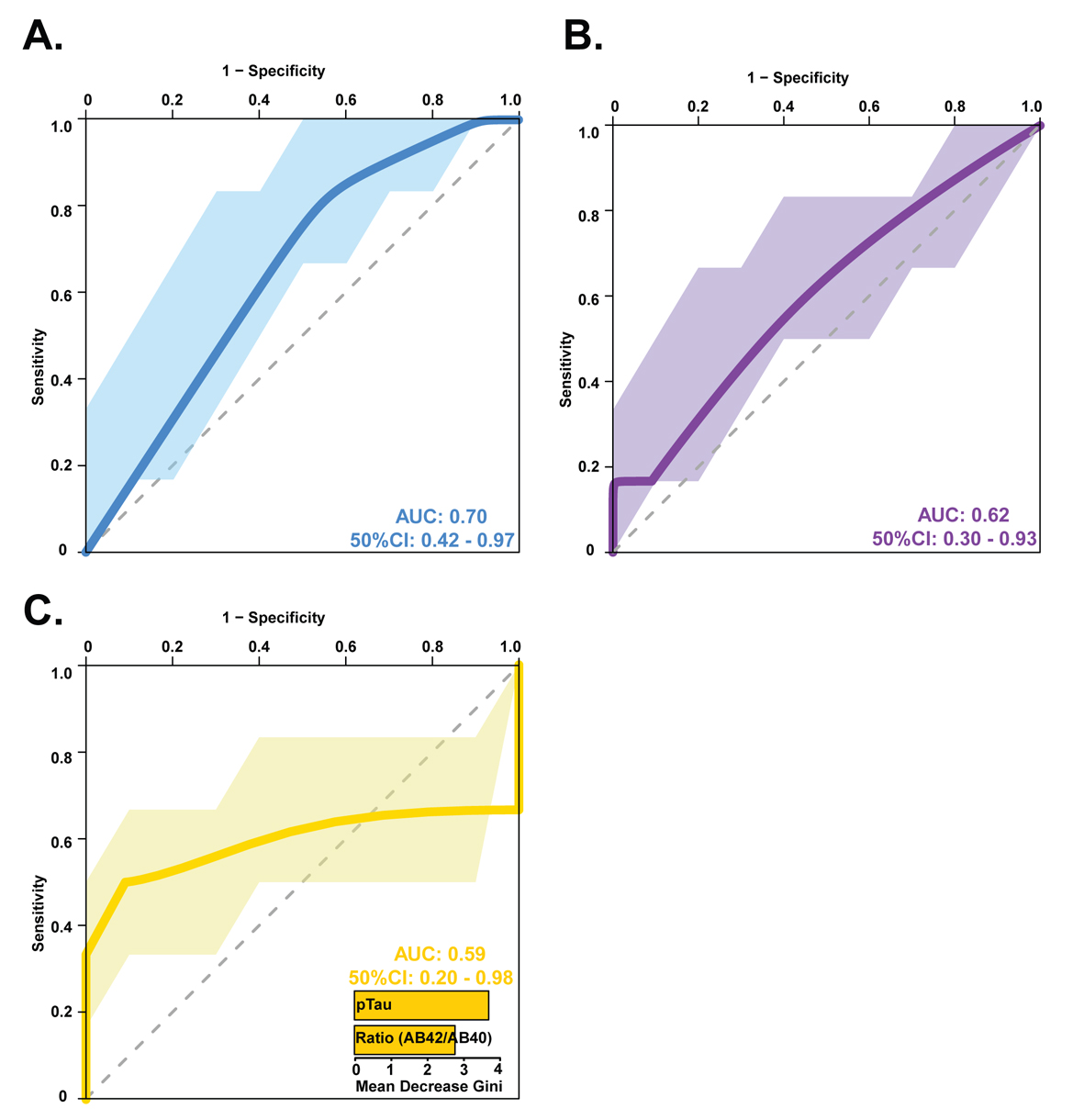


**Fig S1. Performance evaluation of miRNA and combined mi/piRNA signature on DCN cohort. A.** AUC of 0.70 was obtained when the miRNAs signature is evaluated on DCN cohort suggesting decent predictive abilities from mciStable to mciAD. **B.** AUC of 0.62 was obtained when the sncRNAs signature is evaluated on DCN cohort suggesting poor predictive abilities from mciStable to mciAD. **C.** AUC of 0.59 was obtained when pTau and Ab42/40 ratio was used to predict conversion of mciStable to mciAD.
