# Supplementary material for: A combined miRNA–piRNA signature to detect Alzheimer’s disease
Source: Transl Psychiatry. 2019 Oct 7;9:250. doi: 10.1038/s41398-019-0579-2 (PMC6779890; doi:10.1038/s41398-019-0579-2)
Supplement: Supplementary file 3 — Supplemental table 2 [file 41398_2019_579_MOESM3_ESM.pdf]

piRNA	Annotation	Symbol	Gene Chr	Gene Start	Gene End	Gene Length	Gene Strand	GeneId	TranscriptId	Distance to TSS	Ensembl Gene ID	Official Full Name
hsa_piR_019324|chr5:71851020-71851050|+	Distal Intergenic	CARTPT	chr5	71014990	71016875	1886	+	9607	uc003kbv.2	131858	ENSG00000164326	CART prepropeptide
hsa_piR_019949|chr11:122559947-122559977|+	Exon (uc021qrq.1/uc021qrq.1, exon 1 of 1)	UBASH3B	chr11	122526398	122685187	158790	+	84959	uc001pyi.4	-95713	ENSG00000154127	ubiquitin associated and SH3 domain containing B
hsa_piR_020364|chr10:72972395-72972420|-	Distal Intergenic	PLA2G12B	chr10	74694938	74714510	19573	-	84647	uc001jtf.1	-17668	ENSG00000138308	phospholipase A2, group XIIB
hsa_piR_020364|chr14:102317092-102317117|+	Promoter (<=1kb)	ZNF839	chr14	102783714	102809511	25798	+	55778	uc001ylo.3	-260	ENSG00000022976	zinc finger protein 839
hsa_piR_020364|chr17:8227039-8227064|-	3' UTR	LINC00324	chr17	8123948	8127361	3414	-	284029	uc002gkp.4	-3021	ENSG00000178977	long intergenic non-protein coding RNA 324
hsa_piR_020364|chr2:50936204-50936229|-	Intron (uc021vhg.1/9378, intron 21 of 22)	NRXN1	chr2	50145643	51256332	1110690	-	9378	uc021vhg.1	92989	ENSG00000179915	neurexin 1
hsa_piR_020364|chr6:27177263-27177288|-	Exon (uc021ypd.1/uc021ypd.1, exon 1 of 1)	HIST1H2BK	chr6	27106072	27114637	8566	-	85236	uc003nix.2	-30430	ENSG00000197903	histone cluster 1, H2bk
hsa_piR_020364|chr6:27237619-27237644|-	Exon (uc021ypk.1/uc021ypk.1, exon 1 of 1)	POM121L2	chr6	27276842	27280011	3170	-	94026	uc011dku.1	74612	ENSG00000158553	POM121 transmembrane nucleoporin-like 2
hsa_piR_020364|chr6:27293892-27293917|+	Exon (uc021ypq.1/uc021ypq.1, exon 1 of 1)	VN1R10P	chr6	27292540	27293741	1202	+	387316	uc010jqt.4	-30844	ENSG00000220758	vomeronasal 1 receptor 10 pseudogene
hsa_piR_020364|chr6:26554122-26554147|+	Exon (uc021ynp.1/uc021ynp.1, exon 1 of 1)	HMGN4	chr6	26538572	26547164	8593	+	10473	uc003nig.3	15779	ENSG00000182952	high mobility group nucleosomal binding domain 4

Supplemental Table 2: A detailed annotation of the signature piRNAs and their assocation with the Alzheimer's disease
